# Innervation of the Uterus and of Its Vessels

**Published:** 1879-01

**Authors:** 


Innervation of the Uterus and of Its Vessels.—A communication is made on the *Wiena Med. Jahrbucher* by v. Basch and Hoffman, giving the results of numerous experiments they have performed on dogs. They find that the uterus receives its motor fibres from two sources. On the one hand, from the hypogastric nerves proceeding from the posterior mesenteric ganglion; and, secondly, from nerve-fibres issuing from the sacral plexus. It is well known that Spiegelberg denied any motor power to the hypogastric branches, and Frankinhauser, considered that the sacral branches were destitute of motor power. Now, according to the remarks of Basch and Hoffmann, a very distinct antagonism exists between these two sets of nerves. If the hypogastric be electrically stimulated, contraction of the circular fibres of the uterus takes place, the cervix descends in the vagina, whilst the os opens. On the other hand on stimulation of the sacral nerves the longitude fibres are made to contract, the uterus becomes shorter, and the os remains closed. Suppression of respiration, or stimulation of the sciatic nerve, acts in a reflex manner, chiefly on the hypogastric nerves. Their experiments further showed that the vessels of the uterus obtain their nerves from the same sources as the muscular tissue, the nervi hypogastrici supplying the constructing and the sacral nerves the dilating fibres, which can likewise be brought into action reflectorially through the sciatics.—*Lancet*, Nov. 16, 1878.

